# Augmenting research consent: should large language models (LLMs) be used for informed consent to clinical research?

**DOI:** 10.1177/17470161241298726

**Published:** 2024-12-08

**Authors:** Jemima W Allen, Owen Schaefer, Sebastian Porsdam Mann, Brian D Earp, Dominic Wilkinson

**Affiliations:** Monash University, Australia; University of Oxford, UK; National University of Singapore, Singapore; University of Copenhagen, Denmark; University of Oxford, UK; University of Oxford, UK; National University of Singapore, Singapore; National University of Singapore, Singapore; Monash University, Australia; Oxford University Hospitals NHS Foundation Trust, UK; Murdoch Children’s Research Institute, Australia

**Keywords:** Artificial intelligence, clinical research, informed consent, large language models

## Abstract

The integration of artificial intelligence (AI), particularly large language models (LLMs) like OpenAI’s ChatGPT, into clinical research could significantly enhance the informed consent process. This paper critically examines the ethical implications of employing LLMs to facilitate consent in clinical research. LLMs could offer considerable benefits, such as improving participant understanding and engagement, broadening participants’ access to the relevant information for informed consent and increasing the efficiency of consent procedures. However, these theoretical advantages are accompanied by ethical risks, including the potential for misinformation, coercion and challenges in accountability. Given the complex nature of consent in clinical research, which involves both written documentation (in the form of participant information sheets and informed consent forms) and in-person conversations with a researcher, the use of LLMs raises significant concerns about the adequacy of existing regulatory frameworks. Institutional Review Boards (IRBs) will need to consider substantial reforms to accommodate the integration of LLM-based consent processes. We explore five potential models for LLM implementation, ranging from supplementary roles to complete replacements of current consent processes, and offer recommendations for researchers and IRBs to navigate the ethical landscape. Thus, we aim to provide practical recommendations to facilitate the ethical introduction of LLM-based consent in research settings by considering factors such as participant understanding, information accuracy, human oversight and types of LLM applications in clinical research consent.

## Introduction

Artificial intelligence (AI) is transforming various aspects of clinical research, from augmenting participant selection processes (Yuan et al., 2023) and automating clinical data analyses (AlShehri et al., 2024), to assisting in academic writing (Hosseini et al., 2023). Following recent advances in generative AI technology, exemplified by OpenAI’s ChatGPT (Kung et al., 2023), researchers are now considering using such systems based on large language models (LLMs) to enhance the informed consent process in clinical research (Savage et al., 2024). This follows similar proposals to use LLMs to improve consent-taking for medical procedures as part of standard patient care (Allen et al., 2024).

While there is one previous study which claims to use an LLM to obtain consent from prospective participants for clinical research (Savage et al., 2024), in fact this model is based on a rule-based system with fixed responses which does not utilise the full capabilities of generative AI to adapt dynamically to individual participant responses and concerns. Therefore, an actual prototype of an LLM for clinical research consent (CRC) is yet to be developed and trialled, though similar models have been proposed for patients’ consent in clinical practice (Aydin et al., 2023).

Such a tool could theoretically offer substantial benefits both to participants and researchers. As explored in the hypothetical case of Ari (Box 1), LLMs could tailor the information necessary for informed consent to participants’ specific questions and concerns to improve their understanding and engagement in the consent process (Aydin et al., 2023).

Like other forms of digital consent, such as e-consent (Chimonas et al., 2023), LLMs could increase access to relevant information (i.e. beyond a static form) through online user interfaces and visual aids. Additionally, LLMs can use text-to-speech functions (i.e. converting responses created by LLMs from written text into spoken words) and teach-back systems (i.e. providing information and then asking the user to explain it back to check understanding) (Jamerson and Shuster, 2023), as well as translate responses into multiple different languages (Zhu et al., 2023). Not only could these features improve access to the relevant information for people with different learning styles or cognitive disabilities, but it could also enhance accessibility for all participants by offering more ways to engage with the relevant information for decision-making. Moreover, participants can access LLM-based consent processes at their own pace and for extended periods, in contrast to traditional consent conversations which may be constrained by a researcher or clinician’s busy schedule or other time limitations. For researchers, LLM-based consent could streamline administrative processes, improving time- and cost-efficiencies (Tailor et al., 2024). LLM-based consent would also leave a log or transcript of consent interactions, which could be used to document actual information conveyed and/or as reference material for participants and researchers to review the consent process.

However, despite its clear theoretical benefits, LLM-based consent also carries significant theoretical risks and practical challenges. These risks range from technical issues such as the potential for the LLM to provide inaccurate or false information (often referred to as ‘hallucinations’) to ethical concerns, including the possibility of participant manipulation, deception or coercion by the LLM (either deliberately as part of its explicit programming, or inadvertently through incidental biases and misrepresentation within its training datasets).

There may also be challenges relating to accountability, since it would be impossible to pre-approve every individualised LLM response in advance, and the opaque nature of AI systems in general could make it difficult to audit their internal decision-making processes. Furthermore, the use of LLMs could lead to ‘click-through consent’, where participants may skim over critical information without truly engaging. This phenomenon has also been observed in other forms of digital consent, such as e-consent (Chimonas et al., 2023).

Another practical concern is the inability of LLMs to pick up on non-verbal cues, which can be essential in assessing whether a participant truly understands the information or is providing voluntary consent. While computer vision AI software could potentially address this issue by interpreting emotional expressions (Ballesteros et al., 2024), this may introduce further complexity to the AI system and may not fully resolve these concerns.

Additionally, given the stringent requirements for approval of consent processes by institutional review boards (IRBs) (University of Oxford, 2021), LLM-based consent would likely require significant regulatory reform before it could be implemented into practice. These reforms may also vary depending on how researchers intend to integrate LLMs into the existing process of CRC.

Typically, CRC comprises two components: (i) a written participant information sheet (PIS), whether a pen-and-paper version or presented on a computer screen, and (ii) a conversation between a researcher and prospective participant to explain the details of the study and to witness signing of the informed consent form (ICF) (although, in some cases, consent may be obtained purely verbally). Not only do these components serve different purposes in CRC, but they are also subject to different requirements for IRB approval. For example, the content and structure of consent conversations are largely left to the discretion of individual researchers (Davies et al., 2024), while the IRB approval process focuses on the supporting consent documents (i.e. PIS and ICF) (University of Oxford, 2021).

In the next section of this paper (part B), we explore the potential ethical implications of LLM-based consent for clinical research. In part C, we consider the role of IRBs in evaluating the various ways in which an LLM consent model (once developed) could be applied to clinical research. We propose five possible approaches: (1) LLMs as a supplement to existing consent processes, (2) LLMs to replace physical PIS, (3) LLMs to replace the conversation with a researcher, (4) LLMs to replace both components and (5) a hybrid approach of all four options. Finally, we offer several recommendations for researchers and IRBs when considering the future application of LLM-based consent into practice.

For the purposes of this paper, we will restrict our discussion to generative AI models (e.g. LLMs), which are distinct from rule-based chatbots (Savage et al., 2024) or other forms of e-consent (Chimonas et al., 2023). We will explore the use of LLMs only for seeking and obtaining informed consent from prospective participants in clinical research. Therefore, their use in other decision-making tasks in the CRC process, such as selecting eligible participants (Yuan et al., 2023) or as an adjunct tool to assist IRBs in evaluating research protocols (Kannan and Gowri, 2024), is outside the scope of this paper. We will also set aside other, more general ethical issues relating to CRC, such as obtaining consent in situations where the participant may lack decision-making capacity (e.g. in emergency or critical care situations, or participants with intellectual disabilities), and assume that LLMs will be applied to consent processes with a competent adult participant. (There are interesting questions about expanding the application of LLMs in CRC to some of these more ethically nuanced cases, but we will not discuss those here.) We will also set aside wider issues pertaining to the use of LLMs in medicine more generally, such as concerns about information privacy and data security, as well as the impact of biased or missing training data leading to unfair distribution of harms among participants. While these concerns are important, the focus of this paper is to explore the ethical concerns specific to the use of LLMs to seek participants’ informed consent for clinical research.

## Should LLMs be used in CRC?

One of the primary criticisms of current CRC processes is that stringent IRB approval requirements for consent materials fail to promote genuine participant understanding and engagement. Instead, these requirements often create extensive administrative burdens on researchers and IRBs without actually fulfilling (or without fulfilling very well or reliably) the key moral purpose of informed consent (Grant, 2021).

As noted above, LLMs offer immense potential to improve existing processes. However, their use in CRC is not without risk. Before researchers can consider adopting LLM-based consent processes, their use needs to be ethically justifiable to ensure systematic acceptance among researchers, regulators, institutions and the broader public.

### Accuracy, reliability and hallucinations

LLM-based consent processes will likely involve a trade-off between the strict language controls for consent materials enforced by IRBs and the more flexible approach to language generation offered by LLMs. While LLMs can create personalised responses to participants’ enquiries, there is also a risk that the information LLMs provide could be misleading or false (e.g. due to so-called ‘hallucinations’), or conveyed in a way or form that is inappropriate (see *Manipulation vs persuasion* section). This, in turn, could under-mine participants’ autonomous decision-making, which requires, at a minimum, that the information conveyed be truthful and accurate.

Some of these concerns may be addressed through technical strategies to minimise the risk of hallucinations and improve the accuracy of LLM-based responses. For example, the LLM could be programmed with fact-checking mechanisms using retrieval-augmented generation (RAG) (Lewis et al., 2020). This technique allows LLMs to access external databases containing study-specific information to validate responses in real-time (these databases could be pre-approved by IRBs). Additional language constraints could also be programmed into the LLM, such as pre-defined response templates or disclaimers (González Corbelle et al., 2022), to ensure compliance with IRB approval criteria and avoid inconsistencies in study-specific terminology.

While developing a new base LLM model for CRC with standardised language controls would theoretically offer the most tailored solution, the practical challenges of such an approach are substantial. Current state-of-the-art LLMs can cost over $100 million to train and require significant computational resources and expertise (Maslej et al., 2023). Moreover, given the rapid pace of AI development, such models risk becoming obsolete within months of their creation. Instead, more feasible approaches could be considered. One option is to fine-tune existing open-source models, such as Meta’s LLaMA, for the specific task of CRC. These fine-tuned models could then be further adapted for specific studies or institutions, or enhanced with retrieval-augmented generation (RAG) capabilities to access verified study-specific information (Lewis et al., 2020). Alternatively, smaller, specialised language models could be created specifically for CRC tasks, which, while less capable than full-scale LLMs, would be more cost-effective and easier to update (Schick and Schütze, 2020).

However, even with specific language controls, LLM-based consent could lead to inconsistencies due to the personalised nature of the information provided to participants. For example, the LLM could simply fail to mention certain key information about the study if it does not appear during the participant’s consent interaction. This issue could be addressed by ensuring that the LLM is embedded within a broader consent-taking e-platform that has certain fixed parameters for consent information, including at least one mention of all relevant information. Participants could then use the LLM to interact with and elaborate on this information in more detail. As an additional feature, LLMs could also be programmed to generate *personalised* participant information sheets, which summarise the participant-LLM consent interaction and include any additional required information that was not elaborated on.

Finally, LLMs could provide participants with inaccurate or false responses when ‘clarifying’ information, and thus undermine the validity of participants’ consent. For this reason, even if an IRB-approved LLM were developed, it would be essential to ensure appropriate human-in-the-loop processes in the potential case of error or system failures (see *Accountability* section). Over time, human oversight of LLM-based consent may reduce as public trust and expert confidence in the system’s performance increases.

Though technical solutions may help to address concerns about the risk of hallucinations in LLM responses, it is worth noting that no LLM has been successfully developed to generate information sheets that are legally compliant with IRB language criteria (Raimann et al., 2024). Given the generative nature of LLMs, it may be impossible to restrict language generation to a level that is consistent with IRB approval criteria while still maintaining a personalised and interactive user experience. In this respect, it may be easier for researchers to use traditional rule-based e-consent models with only pre-programmed responses, rather than attempting to code LLMs to meet IRB-approved phrasing requirements (Chimonas et al., 2023).

However, if the intention of the consent process is to ensure participants’ willing and informed participation in clinical research, and therefore to respect individual autonomy, one question is whether strict IRB language controls fulfil this goal? This issue may be particularly pertinent if using predictable rulebased systems comes at the cost of improved participant understanding and engagement using LLM-based consent models. Many IRBs may impose these strict controls pursuant to regulatory requirements; however, it is not clear whether current consent elements (e.g. dense, hard-to-read PIS) meet *ethical* requirements for giving valid informed consent (Grant, 2021). Therefore, regulatory reform may be needed to accommodate the more flexible approach to language generated by LLMs.

Indeed, IRBs may accept a more lenient approach to language controls if LLMs are used to augment (or replace) conversations with a researcher rather than the PIS, as these conversations are currently not standardised or reviewed by IRBs (see section C). There are, however, other ethical concerns if LLMs were used to replace conversations with researchers (e.g. a risk of overlooking non-verbal cues which may indicate participants’ confusion, hesitancy or disinterest; the potential impact on public trust) (see *Trust* section). Thus, this trade-off between language controls and personalised information may depend on *how* researchers choose to implement LLMs in CRC, rather than representing a *prima facie* reason to reject LLMs for this use.

### Manipulation versus persuasion

The ability of LLMs to tailor their responses may (inadvertently or intentionally) manipulate, deceive or coerce participants, and thus undermine consent validity. Though an LLM itself has no inherent reason to coerce participants (as a machine algorithm), human developers or implementers may program LLMs for their own purposes (e.g. pharmaceutical companies may seek to subtly coerce or unduly influence individuals to enrol in a particular drug trial) (Bard, 2023).

Concerns about the manipulative potential of LLMs (and/or their programmers or designers) are not unfounded. Several studies highlight LLMs’ ability to adapt responses based on users’ emotions or previous interactions (Carrasco-Farré, 2024; Li et al., 2023; Peng et al., 2024; Wang et al., 2023). While this function allows for more personalised and engaging consent interactions, it may (inadvertently) influence participants’ decision-making processes, often without individuals’ specific awareness (Matz et al., 2024; Ye et al., 2024a). Thus, some have called for an immediate ban on the use of LLMs for CRC, citing that AI’s ability to influence participants’ decision-making is ‘incompatible with the kind of voluntary consent’ required for ethical research (Bard, 2023).

On the one hand, it may be argued that some degree of rational persuasion in LLM-based responses may be ethically permissible to support meaningful participant decision-making (Savulescu and Momeyer, 1997). For example, LLMs could be used to guide prospective participants towards making choices that align with their own values and long-term goals (i.e. ‘beneficent persuasion’) (Giubilini and Savulescu, 2018; Swindell et al., 2010), thus enhancing informed decision-making and individual autonomy.

On the other hand, there is a risk that the use of LLMs may unduly influence or pressure participants’ decision-making, and thus undermine the voluntariness of their consent. For example, the use of LLMs to generate human-like responses may be seen as manipulative, as it induces a perception of human qualities which the LLM does not possess (Sparrow, 2002). Even with a disclaimer acknowledging the use of AI, LLM-based interactions may still unduly influence participants’ decision-making (since manipulation does not always require deception) (Luo et al., 2019).

One approach to address these concerns could be to program the degree of persuasiveness of LLMs (Ekpo, 2023), though determining the bounds of acceptable persuasion in CRC may be ethically challenging. Indeed, human researchers are permitted a certain degree of persuasion during consent conversations (e.g. use of vivid imagery, emotionally charged language, and, in some instances, offering participants compensation) (Nelson and Merz, 2002).

As a rule of thumb, LLM-based consent should be no more or less persuasive than human consent processes. However, the distinction between ethically permissible persuasion and ethically fraught coercion may be practically difficult for researchers to delineate in LLM-based responses (Valenti and Giacco, 2022). For one thing, what might be seen as non-coercive for some participants may be coercive for others, and vice versa. It may also be challenging to objectively measure the coercive effects of LLMs, since enrolment rates may be affected by other confounding factors (e.g. scalability of recruitment process, ease of access to consent information, staff availabilities etc.) and participants may not be fully aware of LLM-based influences on their decision-making. Yet, it is also difficult (or potentially impossible) to monitor the level of coerciveness of human researchers in consent conversations. Thus, at least the persuasiveness of LLMs in consent interactions could be standardised (through programming) and monitored (by researchers via consent transcripts), whereas this is not really possible with human consent conversations.

There may also be ethical concerns if LLMs were used to seek consent for research involving participant deception or incomplete disclosure. While deception in research may be ethically permissible under certain circumstances (e.g. if it is the only way to achieve meaningful research outcomes and the benefits to future patients outweigh participants’ autonomy), it is unclear how people might react to deceptive methodologies which involve LLM-based consent. Further empirical evidence of actual LLM-based consent is needed to address these concerns. It would also be important to consider the degree of deception, since this may influence public acceptance and therefore, whether it is practically feasible to use LLM-based consent in such circumstances. For example, people may be more accepting of LLM-based consent for a study that uses placebos, but not accepting of their use in a study involving deliberate misinformation about essential elements of the study’s procedure.

### Accountability

In general, we recommend that the use of LLM-based consent should not change accountability standards for research. In other words, researchers should remain fully accountable (to the extent that they are if they seek consent themselves) for consent processes, even if the LLM goes awry. This standard for accountability would require more than simply programming LLMs to provide accurate and complete information, but also that LLM-based consent does not compromise participants’ voluntary involvement in research (e.g. by coercing them or failing to pick up on non-verbal cues indicating confusion or hesitancy).

Consequently, a high standard for accountability may deter researchers from adopting LLM-based consent processes if they lack confidence in the technology’s ability to obtain valid consent. On the one hand, if researchers’ scepticism is justified, then LLMs should not be used in consent in the first place. However, if there is evidence demonstrating the validity of LLM-based consent, then doubts about its functioning may be alleviated. Auditable consent transcripts may also mitigate these concerns. Additionally, developers of LLM-based consent models should be responsible for any design failures or technical malfunctions that may arise, and IRBs should ensure that the overall consent process is carried out appropriately.

In addition, most jurisdictions legally require all research consent materials to be approved in advance by an IRB (University of Oxford, 2021). However, given that LLMs are designed to generate personalised responses to user inputs in real-time, it would be impossible for IRBs to pre-approve LLM-based consent interactions. Thus, most jurisdictions would likely require legal reform in order to integrate LLMs into CRC. This raises challenges regarding the accountability of LLM-based consent.

One way to address accountability concerns is to implement human-in-the-loop processes to monitor and review LLM-based consent interactions. This process would require researchers to observe consent interactions in real-time (intervening if the LLM produces content outside IRB pre-approved content guidelines) and to review post-interaction consent transcripts for quality assurance.

However, increasing human oversight could also counteract many of the efficiency gains proposed by using LLMs in CRC in the first place (i.e. if researchers are required to review every LLM-based consent interaction, as well as correct any misinformation it provides participants).

There may be several strategies to offset the human-oversight burden. For example, researchers could select a random sample of LLM-based interactions to audit (rather than auditing all interactions). LLMs could also be programmed to self-evaluate interactions and flag particular cases of concern (e.g. by identifying specific keywords or phrases in participant responses that might indicate confusion or hesitancy), thus prompting further review and clarification by a researcher.

Yet, as researchers are progressively taken out of the loop, concerns about accountability become more prominent. Ongoing research will likely be needed to determine an appropriate balance between the efficiency of LLM-based consent and the need for sufficient human oversight to ensure participant safety. Future research should also consider the ethical ramifications of complete absence of human-in-the-loop processes in CRC.

It may be possible, at least at the outset, to introduce a no-fault compensation to address some of these accountability concerns. So, if something goes wrong with the LLM specifically in the consent-taking process, there could be compensation for participants with no need to establish who was at fault (e.g. LLM developers, private companies, researchers, research institutions). While individual claims would not be subject to fault verification, the overall process could then be monitored and decisions about accountability could be made accordingly (e.g. based on the rate and type of errors encountered). This approach may provide reassurance for some researchers and participants who may be initially hesitant to uptake LLM-based consent processes due to these concerns.

### Trust

CRC is important not only for establishing participants’ understanding of the relevant information for informed decision-making, but also to ensure their willing participation and suitability for research. LLMs’ inability to pick up on participants’ non-verbal cues could result in a failure to recognise when a participant is confused, disinterested or hesitant to enrol in a study. For researchers, this may be problematic if LLMs fail to adequately address participants’ concerns, leading to reduced enrolment rates. For participants, failure to recognise non-verbal cues may undermine their autonomy and trust in research enterprise.

While it may be possible to program LLMs to recognise certain facial expressions or patterns of behaviour (using visual interfaces) (Ballesteros et al., 2024), it is also likely that human processes will still be required to pick up on these nuances. Therefore, maintaining some degree of human involvement, regardless of the extent to which LLMs are applied to CRC, will be integral to ensure public trust in research, and therefore the successful integration of LLMs.

Finally, even if LLMs in CRC are legally and ethically acceptable, public mistrust in LLMs could undermine the legitimacy of their use. For example, public concerns about the transparency and accountability of LLMs in handling personal data could lead to resistance or outright rejection of their use in CRC, similar to the backlash seen in other data-intensive health research initiatives (Xafis et al., 2019). Thus, social license, which involves earning the public’s and stakeholders’ trust in a given process, will be incredibly important when considering implementing LLM-based consent (Muller et al., 2021). In particular, LLM-based consent should not only comply with existing regulations, but it should also align with public expectations of CRC in general to ensure both the legitimacy of its processes and to minimise the risk of public criticism. In addition to addressing the ethical concerns already mentioned in this paper, strategies to achieve social license for LLM-based consent may involve proactive public engagement, participant feedback forms, adherence to privacy standards and transparent communication about participants’ data usage and protection (Muller et al., 2021).

Naturally, social license will be contextual not only across societies, but also across time: as more people become comfortable using LLMs, obtaining social license for their use in CRC will become easier. In some ways, this may suggest that a ‘slower’ approach to integrating LLMs in CRC is preferable, in tandem with growing public acceptance of LLMs in general.

## If so, *how* should LLMs be applied into practice?

Notwithstanding the ethical concerns raised in the previous section, ultimately, the justification for the use of LLMs in CRC will likely depend heavily on *how* researchers intend to integrate these tools into existing consent processes.

We suggest five possible approaches to the use of LLMs in CRC (detailed below). When evaluating each of these approaches, we consider the following criteria:

- *Accuracy and utility of information*: Ensuring participants receive accurate and complete consent information that is useful for their decision-making.
- *Participant autonomy*: Evaluating how well each approach supports individual autonomy and decision-making.
- *Personalisation*: Assessing the ability of the model to tailor information to the needs of each participant.

*Accountability and transparency*: Considering whether the approach allows for auditable and transparent consent processes.

- *Feasibility of integration into existing research frameworks*: Evaluating the practical ease of integrating each model with current regulations and practices, including IRB approval and researcher involvement.

Box 2 summarises the ethical evaluation of the five proposed approaches for integrating LLMs into CRC according to the above criteria. This comparison allows for a clearer understanding of how different LLM-based consent processes balance ethical considerations and practical implementation. Box 3 outlines the benefits, challenges and relevant recommendations for implementation of each of the proposed approaches.

### As a supplementary resource to existing consent processes

At least initially, it seems likely that LLMs could be used as a supplementary resource to augment existing consent processes by improving participants’ understanding and engagement. In this approach, participants would continue to have conversations with researchers, and still receive detailed written PIS. For IRBs, this approach may seem the least problematic (of the five listed) as it maintains the integrity of accepted practices in CRC, while also leveraging the advantages of AI technology (i.e. personalised interactions, flexible access to information and improved engagement through multimedia functions, simplified language and translation tools).

However, there may still be concerns with even this most minimal LLM-as-a-supplement approach. It would therefore be essential that LLMs are programmed to recognise the limits of their capabilities, and to refer users to human researchers when questions exceed the AI’s scope of knowledge or when a human-based approach is required. Initially, it may also be necessary for a researcher to review interactions with the LLM so that they can troubleshoot technical errors or intervene if the LLM goes off track. Researchers (and participants) would also need to upskill and train to use LLM-based consent models.

Thus, using LLMs as a supplementary resource may score highly with regards to feasibility as it maintains traditional processes and minimises the risk of disrupting existing frameworks. This approach also ensures accuracy by allowing researchers to review interactions with the LLM. However, the trade-off may lie in participant autonomy, as participants would still need to arrange a time to meet with researchers and would have limited flexibility to navigate the consent process at their own pace and convenience.

### To replace the physical PIS

Participant information sheets (PIS) have been heavily criticised for failing to adequately promote participant autonomy due to information overload and depersonalisation of the consent process (Grant, 2021). These documents are often complex (Fischer et al., 2021; Ménoni et al., 2010; Taylor and Bramley, 2012), lengthy (Emanuel and Boyle, 2021) and inaccurate or out-dated (Luehnen et al., 2018). They also fail to adjust to participants’ individual informational needs, ultimately creating a ‘one-size-fits-all’ approach to consent (Habiba et al., 2004). Consequently, participants often view the consent process in clinical research as intended to protect researchers from legal liability, rather than support participants to understand the necessary information for autonomous decision-making (Chen et al., 2020).

Despite these shortcomings, researchers are often legally compelled to include highly detailed PIS during the consent process to comply with strict IRB approval requirements. These requirements also mandate the exact phrasing used when providing information to participants (University of Oxford, 2021).

Replacing traditional PIS with LLMs would address some of these challenges by providing participants with tailored, real-time information specific to their questions and concerns, aligning well with the criteria of personalisation and autonomy. LLMs would also offer a more interactive and engaging platform than static paper-based PIS for participants to ask questions and receive clarifications in real-time.

However, this approach would also require significant regulatory reform, therefore raising concerns about accountability if stringent IRB regulations are required and the feasibility of replacing legally mandated physical documents with dynamic, AI-driven interactions. In particular, IRBs would need to clarify with existing regulations whether LLMs should uphold the same stringent language criteria required of PIS. This reform would involve expanding research codes of conduct to explicitly allow LLM-based consent processes as an alternative to written PIS.

Additionally, research regulations typically require that participants are provided with a take-home copy of the PIS and/or ICF. In the case of LLM-based consent, this requirement could be met by allowing participants continual access to LLM interactions and previous consent transcripts.

As noted above, if language requirements for LLM-based interactions are lenient, IRBs could approve a ‘base’ script of a typical consent interaction which the LLM could tailor to participants’ specific questions and concerns. In this approach, LLMs would also need to be programmed with a core set of minimum information to ensure participants are appropriately informed prior to giving their consent (University of Oxford, 2021). These dynamic scripts could also include conditional logic to adapt responses based on participants’ prior knowledge (Neupane et al., 2024), specific concerns (Ye et al., 2024b) or cultural nuances (Ochieng et al., 2024).

However, if stringent language requirements are enforced, then LLMs may not offer any additional benefit over traditional e-consent processes. In fact, given their use of generative AI, LLMs may present additional challenges for researchers when coding to ensure they do not respond outside the specific IRB-approved phrasing.

Yet, there are several criticisms to the uniform approach to information disclosure currently adopted by IRBs. Firstly, although PIS allows *equal* access to consent information, it may not be *equitable*. For example, some participants may have cognitive or visual impairments which limit their capacity to read and comprehend information via traditional PIS. Additionally, providing participants with PIS does not guarantee that they will actually read the information (indeed, many participants do not) (Agozzino et al., 2019; Özhan et al., 2014). Researchers may still be liable in this case if the participant claims that they were not adequately informed prior to enrolling in the study (University of Oxford, 2021).

Thus, LLMs may allow more equitable access to consent information, while still providing participants and researchers with a detailed reference to validate the information that has been provided during the consent process.

Ultimately, the decision to replace PIS with LLMs comes down to a trade-off between the potential benefits of personalisation offered by LLMs and the need for a standardised and auditable approach to information disclosure.

### To replace the conversation with a researcher

The consent conversation in clinical research aims to enhance participant understanding and engagement, as well as establish participants’ suitability and willingness to participate in research. One possibility is to replace this conversation with an LLM interaction.

Though preliminary evidence focuses mainly on rule-based e-consent models (Bibault et al., 2019; Savage et al., 2024), LLMs could theoretically enhance the potential for participants to comprehend and engage with relevant information over human consent conversations by simplifying complex language, using multimedia elements (e.g. diagrams, audio-visual aids), and creating personalised and interactive user experiences. LLMs could also allow participants to access consent materials remotely, allowing them to review information at their own pace and convenience. Thus, replacing human conversations with LLMs has significant potential to enhance the personalisation of the consent process and participant autonomy.

Since current consent conversations with a researcher are largely undocumented (Davies et al., 2024), LLMs also could enhance the transparency of consent interactions by generating auditable transcripts, and therefore improve accountability of the consent process. This feature may be useful in identifying whether LLM-based responses include inaccurate or manipulative content. Consent transcripts could also be monitored and reviewed by researchers to verify LLM consent interactions and maintain accountability of the consent process. Given appropriate consent, such transcripts could also provide data for further studies on consent interactions.

While LLMs may improve participants’ engagement and understanding through personalised and interactive responses, their inability to recognise non-verbal cues may impact the validity of the consent they obtain. One option could be to program LLMs to identify certain features of participants’ responses that might indicate disinterest, confusion or hesitancy (Peng et al., 2024). However, this approach would likely require some form of human oversight or referral, at least initially, to ensure proper processing of participants’ consent (i.e. that participants are suitable for the study and that their voluntary informed consent has been appropriately obtained). Over time, the amount of human oversight required may reduce, as technology improves and public trust in LLMs increases. Thus, the feasibility of this approach would require balancing the extent to which LLMs are used to provide personalised consent information with sufficient oversight by researchers.

### A combination of (2) and (3)

Replacing conventional methods of informed consent in clinical research with an entirely LLM-based approach is the most intensive of the options presented in this paper. While this approach offers potential benefits in terms of flexibility and personalisation, as participants could engage with the consent process at their own convenience, there may also be significant concerns around accuracy, as LLMs may struggle to consistently provide appropriate, error-free information without human oversight. However, human consent-seeking is not an ideal system for information disclosure, and humans are also prone to errors and biases. Thus, LLM-based consent does not necessarily need to be completely perfect, it simply needs to be as good as (or better than) human processes.

Additionally, the absence of human researchers may alienate participants who are less comfortable with technology or prefer the reassurance of speaking directly with a person. There may also be a risk that the LLM’s responses are overly generalised or fail to grasp the unique concerns or cultural nuances of individual participants. Therefore, personalisation could remain surface-level, addressing basic needs but potentially missing the deeper, relational aspect of informed consent.

Moreover, while there are separate ethical concerns associated with replacing either physical PIS or consent conversations with a researcher, these separate concerns would be compounded if both were replaced by LLMs, and may even be amplified given the complete absence of human processes.

Accountability is another major issue, as removing human researchers eliminates traditional safeguards, and the feasibility of implementing this approach is likely to be low given current technological limitations and the need for regulatory reform. It is consequently unlikely to be acceptable in the medium-term to replace both researcher conversations and PIS with LLM, not only because it is unknown whether existing LLMs have the technical capacity, but also because it is unlikely that there would be sufficient social license for this approach.

### A hybrid approach of all four

**Figure 1:**
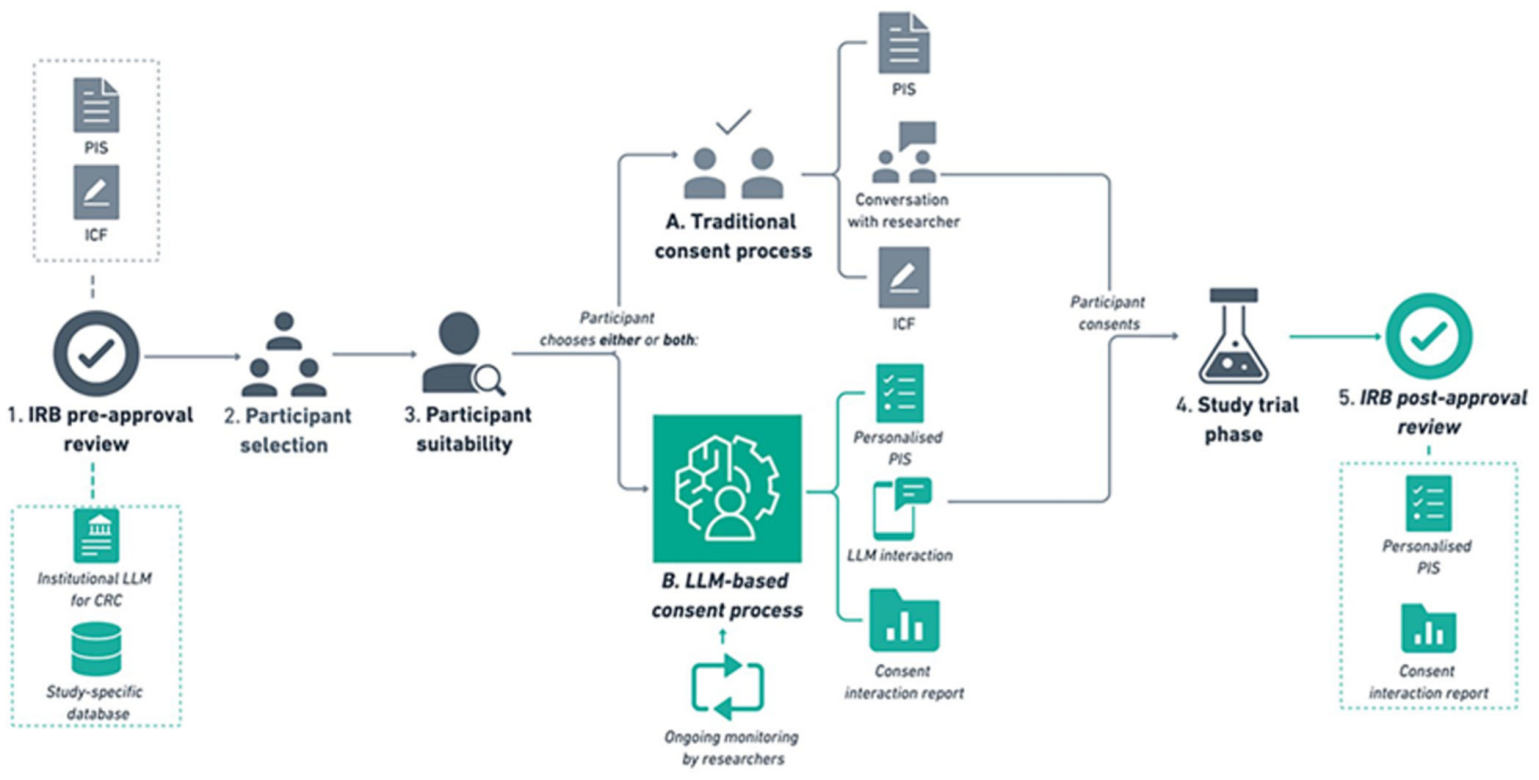
A hybrid approach to the implementation of LLM-based consent in clinical research. This figure provides a visual representation of the hybrid approach to LLM-based consent outlined in section C. The IRB is responsible for reviewing consent materials (traditional consent: PIS and ICF, LLM-based consent: institutional LLM for CRC model programming and study-specific database of consent materials) for pre-approval. Participants undergo a selection process for their eligibility. Their suitability and willingness to participant in the study is checked by a researcher. Participants are then given the choice between undergoing (A) a traditional consent process or (B) LLM-based consent, or both. Researchers are also responsible for the ongoing monitoring of the LLM-based consent process. If the participant consents to the study, they are enrolled and go onto the trial phase of the study. The personalised PIS and consent interaction reports also undergo a follow-up review for post-approval by the IRB. Abbreviations: participant information sheet (PIS), informed consent form (ICF), institutional review board (IRB), large language model (LLM), clinical research consent (CRC).

Ultimately, we recommend a fifth hybrid approach to LLMs in CRC which combines elements of the above options (Figure 1). We believe this hybrid approach offers a practical solution that maximises participant autonomy and personalisation while maintaining accuracy and transparency through human oversight. While it requires considerable hands-on involvement and oversight from researchers and IRBs, this model provides a potential roadmap for developing more efficient, less human-dependent LLM-based consent processes in the future. This hybrid approach is also the one described in Ari’s case from the *Introduction*.

Following an appropriate selection process to determine their eligibility for the study, the prospective participant would have an in-person discussion with one of the study’s researchers. The purpose of this preliminary conversation would be to undertake a surface-level assessment of the individual’s suitability (i.e. willingness to participate in the study). Prospective participants would then have the option to undergo a traditional consent process (i.e. with a physical PIS and an additional conversation with a researcher at a later time to sign the ICF) or an online consent interaction with an LLM (or both).

Indeed, some participants may choose to use both the traditional consent process and LLM-based consent for several reasons. They might wish to preserve the human interpersonal elements offered by a conversation with a researcher while also accessing detailed and tailored consent information from the LLM. Alternatively, some people might want to avail themselves of both consent options to maximise their access to relevant information, or others might want to use both to satisfy themselves that they are not missing out or getting an inferior option by only seeking one approach to consent.

Hence, the hybrid approach is a form of meta-consent, allowing participants to choose their preferred consent process based on their values and interests (Ploug and Holm, 2016). This flexibility respects individual autonomy and acknowledges that participants may have different comfort levels with technology and varying preferences for how they wish to engage with the consent process.

If the individual agrees to the LLM-based consent interaction, the researcher would send them a link to the LLM interface for an in-depth discussion of the relevant information about the study. Individuals who indicate their interest in participating in the study can use the LLM-based platform to sign their consent. Following the interaction, the LLM generates a personalised participant information sheet (PPIS) which would include any information that was not discussed via the LLM-based app. The LLM also creates a consent interaction report for the researcher to review (as required). This report includes information about the duration of the interaction, the number and type of questions that were asked, what the participant’s key concerns were, and any additional considerations flagged by the LLM (e.g. information that might indicate participants’ confusion or hesitation).

Finally, participants could opt-in to undergo follow-up with a researcher to check their understanding and willingness to participate. The LLM-based platform could then be used continuously throughout the trial period to check on the participant’s progress and clarify any additional questions or concerns.

Once the trial period is over, participants could continue to access LLM-based consent to provide them with information about the ongoing use of their data. This feature has been explored in more detail in the context of consent for biobanking (Barnes et al., 2024). By combining LLMs with blockchain technology, sample donors may be able to track how their samples are used in current and future studies and adjust their consent preferences as needed (Barnes et al., 2024).

To review this hybrid process as we have imagined it, IRBs would need to consider the following:

- IRB pre-approval of LLM ‘base’ consent scripts (i.e. minimum information requirements determined by IRB) and training data to ensure accuracy of consent information.
- Ensure appropriate language parameters for LLM responses and content disclaimers.
- Once implemented, the IRB would audit a random sample of consent transcripts and consent interaction reports to validate the LLM’s performance.
- Follow-up with researchers and participants to ensure the consent process is satisfactory and to address any concerns or challenges.

However, a hybrid approach to LLM-based consent would also require the most hands-on involvement from IRBs and researchers of all five approaches, since both traditional and LLM-based consent processes would need to be generated and reviewed by researchers and IRBs, respectively. Thus, a hybrid approach prioritises participant autonomy at the expense of an efficient research ecosystem. However, this approach may offer a pathway to more efficient LLM-based consent processes (i.e. one with reduced human oversight and involvement).

## Practical recommendations

As discussed above, the integration of LLMs into the CRC process presents both opportunities and challenges. The following recommendations outline strategies to effectively implement LLM-based consent systems, ensuring participant understanding and maintaining ethical standards.

Firstly, hospitals and other healthcare institutions should consider adopting ‘institutional’ LLMs to minimise the risk of inaccurate, conflicting or misleading information. These institutional LLMs could be based on either large open-source AI models (like OpenAI’s GPT-4 or Meta’s Llama) fine-tuned on the institution’s specific values and secured behind a digital fire-wall, or they could be based on smaller custom models designed specifically for clinical use within the hospital. (While this second option would be more expensive, it may mean that the LLM is less likely to provide responses outside its specific remit.) Institutional LLMs should also be equipped with language controls and disclaimers about the nature of the consent interactions, as well as based on training data approved beforehand by an IRB, to ensure that the LLM’s responses comply with hospital policies. Institutions should also deploy LLMs within closed networks (whether they are based on larger open-source models or smaller custom ones) to protect participants’ data privacy and security. Institutional LLMs should also be designed with features that enhance participant engagement and understanding, such as multimodal communication (e.g. visual aids, imagery, text-to-speech functions), translation tools and LLM-generated summary sheets that detail the key information and gaps in knowledge. These institutional LLMs could then be trained on study-specific consent materials approved by an IRB to create customised models specific for CRC.

Secondly, LLM interactions should be monitored by human-in-the-loop processes to ensure accuracy and accountability. One possible approach to human oversight could be for researchers to randomly select a sample of LLM consent interactions to review for quality control. As an additional tool, LLMs could self-evaluate their own interactions with participants and generate reports that detail the duration of the interaction, questions or concerns raised by the participant, and any potentially significant features of the interaction for the researcher to review. For example, the LLM could identify potential signs of confusion, disinterest or hesitancy from participants during consent interactions. These detailed consent interaction reports not only provide transparency to IRBs and researchers, but also serve as an auditable record of the consent process. Another important source of human oversight could come from participant feedback forms, which might be used to fine-tune LLMs’ responses and identify particular areas of participant concern. It seems likely that, at least initially, LLM consent interactions will be closely monitored by human researchers, potentially requiring all interactions to be checked first by a human, until there is significant enough evidence to reassure researchers and IRBs of the quality of LLM-based consent. Initial introduction of LLM-based consent processes should also allow participants to have the option to follow up with a researcher after an LLM interaction if they so choose.

Finally, it will likely be necessary to introduce LLMs into CRC in a staged approach, to allow time for both researchers and participants to adapt to a new system. We suggest that our proposed hybrid approach is the optimal way for researchers to maximise the benefits of LLM-based consent processes while minimising the potential risks by maintaining an appropriate amount of human oversight. However, others may choose to adopt a variety of different combinations of the five approaches suggested in this paper.

The success of LLM-based consent will depend heavily on public acceptability and social license of these systems in research settings. Public engagement forums and mechanisms for participant feedback are essential to ensure trust in these systems, alongside stringent adherence to data privacy and security standards. Institutions should also be transparent about how participants’ data is used and stored. Additionally, it may be necessary to adopt regulatory reforms to allow a more flexible approach to the language used in consent processes, particularly if LLMs are used as an alternative to traditional written PIS.

### Impact on key stakeholders

The adoption of LLM-based consent models will impact different stakeholders in unique ways, and understanding these effects is crucial for effective implementation.

For participants, LLMs offer greater personalisation, access to real-time information, and flexible interactions, making the consent process more engaging and tailored to their specific needs. However, concerns about the accuracy and utility of AI-generated information and the loss of human interaction may affect participants’ confidence in these systems to provide fully informed consent. To address this, we recommend features to enhance understanding such as multimodal interaction options (e.g. visual aids, text-to-speech) and clear means for participants to engage with researchers for follow-up discussions, in cases where direct human interaction is preferred.

For researchers, LLMs offer the potential to streamline the consent process, making it more efficient and scalable. This could allow researchers more time for other tasks which specifically require human involvement, such as addressing challenging ethical dilemmas or providing emotional support to participants with sensitive or culturally-nuanced concerns. However, researchers must also navigate the challenge of ensuring the accuracy of the LLM’s responses and maintaining accountability in case of errors. Researchers will need to regularly review LLM-generated interaction reports and monitor a sample of participant interactions to ensure that ethical standards are upheld. These detailed logs of the consent interaction generated by the LLM could provide a useful resource for researchers, both in terms of fine-tuning future LLM-based consent models but also to understand what elements of the consent process individuals have difficulty understanding and what they find most important or interesting. These insights could help refine the way information is disclosed to participants and improve the consent-seeking process overall. Additionally, the hybrid approach we propose provides researchers with a valuable tool, but it also requires their active oversight and intervention when necessary. Thus, consideration should be given as to whether LLM-based consent processes will indeed be cost-efficient, since reviewing LLM consent interactions may increase time commitments for researchers, particularly in larger trials.

For IRBs, the primary concern is ensuring that LLM-based consent processes comply with regulatory and ethical standards. While LLMs could improve the transparency of the consent process through auditable transcripts and real-time monitoring, the risks of manipulation, misinformation, or a lack of standardisation in responses may raise concerns. IRBs would need to approve LLM training data, consent scripts, and ensure that language restrictions and disclaimers are embedded in the system to maintain consistency of information. A phased introduction of LLM-based systems, coupled with random audits of LLM interactions, would allow IRBs to manage these risks while benefiting from the enhanced oversight that AI-based systems could offer.

### Recommendations for further empirical research

Further empirical research is needed to evaluate the real-world effectiveness of LLM-based consent models and guide regulatory reforms necessary for their adoption into practice. Studies should explore how LLMs affect participant understanding and retention of consent information, and whether they can make the consent process more engaging through interactive features such as adaptive questioning, tailored visual aids and real-time feedback. Consideration should also be given to assess the effectiveness of personalised or persuasive techniques employed by LLMs to enhance recruitment strategies and participant commitment to research projects. It will also be important to assess how well LLMs function across diverse participant groups and to investigate the scalability of LLM-based consent processes for larger clinical trials or across multiple research sites.

## Conclusion

LLM-based consent in CRC presents a promising opportunity to enhance participant understanding and engagement, and streamline consent processes to improve scalability and cost-effectiveness of clinical research studies. However, the successful integration of LLMs in CRC requires careful consideration of the potential ethical risks.

In this paper, we have reviewed the key risks related to accuracy, manipulation, the impact on public trust, and accountability, and considered ways to address these. We have argued that the weight of these concerns depends on how LLM are implemented into the research consent process and considered several different approaches to the integration of LLMs in CRC. Ultimately, we have suggested that a hybrid model may provide an attractive way to realise the benefits of LLM-based consent, while maximising participant autonomy and practical safeguards. More evidence based on actual LLM-based consent models is needed to validate the real-world effectiveness of these models in consent. Additionally, depending on the approach to integration, LLM-based consent may require regulatory reforms before it can be used in practice.

## Box 3 Summary of the theoretical benefits, theoretical challenges and practical recommendations for each approach to LLM-based CRC

**Table:**

Application of LLM	Theoretical benefits	Challenges and Risks	Recommendations
LLM-as-a- supplement	Easy to implement (requires minimal social license, maintains accepted standard for consent in research).	No (or minimal) efficiency gains (requires additional processes and technical infratructure without reducing workload; researchers and participants required to upskill in technical proficiency) Increased risk of information overload (due to multiple sources of information)	No additional recommendations
LLM replace PIS	More personalised approach to information disclosure	Requires regulatory reform (in most jurisdictions) due to stringent language criteria requiring pre-approval by IRBs	IRBs approve a ‘base’ script of the study-specific consent interaction which includes conditional logic to adapt information based on participants’ prior knowledge and specific concerns IRBs audit a random selection of LLM consent interactions to monitor and review
LLM replace researcher conversation	Improves transparency (consent conversations are not typically reviewed by IRBs)	Inability to assess participants’ non-verbal cues (e.g. confusion, hesitancy, disinterest, involuntary participation) Potentially increased burden on researchers (if expected to review all LLM consent interactions)	Requires anti-manipulation and coercion controls, such as language safeguards and disclaimers stating the role of Al Implement guidance mechanisms within LLM programming to ensure participants make informed decisions that reflect their personal values and long-term goals
LLM replace both PIS and researcher conversation	Potential for fully automated consent process Maximises efficiency Standardisation of consent process	Significant risk of misunderstanding or miscommunication without human oversight Elevated risk of losing nuanced understanding of participants’ needs	Requires IRB pre-approved process for monitoring and contingency plan to address LLM system-failures or participant misunderstandings Establish process for real-time human intervention in case of LLM error
Hybrid approach	Offers flexibility by combining traditional and LLM-based consent processes, catering to different participant needs	Requires significant involvement from IRBs and researchers due to the need to generate, review, and monitor both traditional and LLM-based processes, leading to practical inefficiencies Potential for increased complexity in managing and auditing the dual consent processes	IRB pre-approval of LLM ‘base’ consent scripts and training data. Establish appropriate language parameters and disclaimers for LLM responses Audit a random sample of consent transcripts and interaction reports to validate the LLM’s performance Implement follow-up processes with researchers and participants to ensure satisfaction and address concerns Plan for future reduction in human oversight as the LLM-based system becomes more reliable
